# Efficacy and safety of electroacupuncture with different acupoints for chemotherapy-induced nausea and vomiting: study protocol for a randomized controlled trial

**DOI:** 10.1186/s13063-015-0734-x

**Published:** 2015-05-12

**Authors:** Bo Chen, Shu-xiang Hu, Bao-hu Liu, Tian-yi Zhao, Bo Li, Yan Liu, Ming-yue Li, Xing-fang Pan, Yong-ming Guo, Ze-lin Chen, Yi Guo

**Affiliations:** Acu-moxibustion and Tuina Department of Tianjin University of Traditional Chinese Medicine, Tianjin, 300193 China; Acupuncture Research Center of Tianjin University of Traditional Chinese Medicine, Tianjin, 300193 China; Clinical Practice Teaching Department of Tianjin University of Traditional Chinese Medicine, Tianjin, 300193 China

**Keywords:** chemotherapy-induced nausea and vomiting, acupuncture, matching acupoint, randomized controlled trial

## Abstract

**Background:**

Many patients experience nausea and vomiting during chemotherapy treatment. Evidence demonstrates that electroacupuncture is beneficial for controlling chemotherapy-induced nausea and vomiting (CINV). However, the acupoint or matching acupoint with the best efficacy for controlling CINV still remains unidentified.

**Methods/Design:**

This study consists of a randomized controlled trial (RCT) with four parallel arms: a control group and three electroacupuncture groups (one with *Neiguan* (PC6), one with *Zhongwan* (CV12), and one with both PC6 and CV12). The control group received standard antiemetic only, while the other three groups received electroacupuncture stimulation with different acupoints besides the standard antiemetic. The intervention is done once daily from the first day (day 1) to the fourth day (day 4) during chemotherapy treatment. The primary outcome measures include frequency of nausea, vomiting and retching. The secondary outcome measures are the grade of constipation and diarrhea, electrogastrogram, assessment of quality of life, assessment of anxiety and depression, and other adverse effects during the chemotherapy. Assessments are scheduled from one day pre-chemotherapy (day 0) to the fifth day of chemotherapy (day 5). Follow-ups are done from day 6 to day 21.

**Discussion:**

The aim of this study is to evaluate the efficacy and safety of electro-acupuncture with different acupoints in the management of CINV.

**Trial registration:**

The register number of randomized controlled trial is NCT02195908. The date of registration was 21 July 2014.

## Introduction

### Background

Despite the relevant progress achieved in the last 20 years, chemotherapy-induced nausea and vomiting (CINV) is still one of the most distressing side effects of cancer chemotherapy [1]. As 42 to 52% of patients experience nausea daily during regular treatment, nausea continue to be a significant problem in chemotherapy treatment [2]. *The National Institutes of Health* (*NIH*) 1997 Consensus Statement stated that “promising results have emerged showing efficacy of acupuncture in adult postoperative and chemotherapy nausea and vomiting” [3]. Acupuncture has gained increasing popularity since then as a choice of nonpharmacological adjunctive treatment. Evidence-based medicine demonstrates that electroacupuncture is beneficial towards chemotherapy-induced acute vomiting, and The American Society of Clinical Oncology recommends the usage of electoacupuncture for managing nausea and vomiting in cancer patients [4].

However, no consensus exists on which set of compatible acupoints is optimal, despite trials having been conducted on acupoint matching. Some trials use single acupoints, while others use double acupoints or matching acupoints. For example, Ezzo [5] found that stimulating PC6 may be beneficial for various conditions involving nausea and vomiting, and Shen [6] concluded that electroacupuncture on PC6 (Neiguan) and ST36 (Zusanli) is more effective in controlling emesis than minimal needling or antiemetic pharmacotherapy alone. Hence the answer to the following question remains unknown at present: Which compatible acupoint or acupoints have better efficacy for CINV?

The aim of our study is to evaluate the different efficacies and safety of electroacupuncture on a single point and matching acupoints in the management of chemotherapy-induced nausea and vomiting.

### Hypotheses

According to the theory of Traditional Chinese Medicine (TCM), acupoint compatibility can achieve a synergistic effect. Our prior literature review found that clinically matching acupoints are applied more often than a single acupoint. The pairing of PC6 (Neiguan) with CV12 (Zhongwan) is one of the most commonly used matching acupoints. Our hypothesis is that this pair helps to reduce toxicity and enhance efficacy in the management of chemotherapy-induced nausea and vomiting.

### Objectives

#### Primary objective

Our primary objective in this trial is to assess the clinical efficacy and safety of electroacupuncture on single point or on double points in the management of chemotherapy-induced nausea and vomiting (CINV).

#### Secondary objective

##### Secondary objective

Our secondary objective is to assess the quality of life, anxiety and depression of patients and the other side effects of chemotherapy, such as diarrhea, constipation, *etcetera.*

## Methods/Design

### Trial design

This trial is designed as a four-armed parallel randomized controlled trial.

## Participants

### Eligibility criteria

#### Inclusion criteria

The inclusion criteria include the following:

1. Be diagnosed by histopathology as a tumor patient and accepts chemotherapy.
2. The scoring of Karnofsky (KPS) must be more than 70.
3. Patients of either gender, between 18 to 79 years old inclusive.
4. Patients receiving chemotherapy as outpatients or inpatients.
5. Patients receiving chemotherapy either for the first time or undergoing multiple cycles will be taken into the study only once.
6. Receive cis-platinum (cis-platinum ≥75 mg/m2) or antharcycline combined chemotherapy (doxorubicin ≥40 mg/m2 or epirubicin ≥60 mg/m2)
7. Life expectancy of at least 6 months.
8. Willing to participate in the study and be randomly allocated into one of the four study groups.

#### Exclusion criteria

Exclusion criteria include the following:

1. Receive radiotherapy concurrently with chemotherapy.
2. Patients with gastrointestinal tumors.
3. Liver disease patients with serious complications, or with serious hepatorenal abnormal function (glutamic oxalacetic transaminase (AST), glutamic-pyruvic transaminase (ALT), or total bilirubin (TBIL) three times higher than normal or blood urea nitrogen (BUN) or urine creatinine (Cr) two times higher than normal).
4. Presence of cardiac pacemaker.
5. Active skin infection.
6. Nausea and/or vomiting as a result from opioids or metabolic imbalance (electrolyte disturbances).
7. Patients unable to provide self-care or communicate, or that have mental sickness,
8. Nausea and/or vomiting resulting from mechanical risk factors (that is, intestinal obstruction).
9. Brain metastases or intracranial hypertension.
10. Women who are pregnant and or lactating.

### Settings and data collection

**Figure 1:**
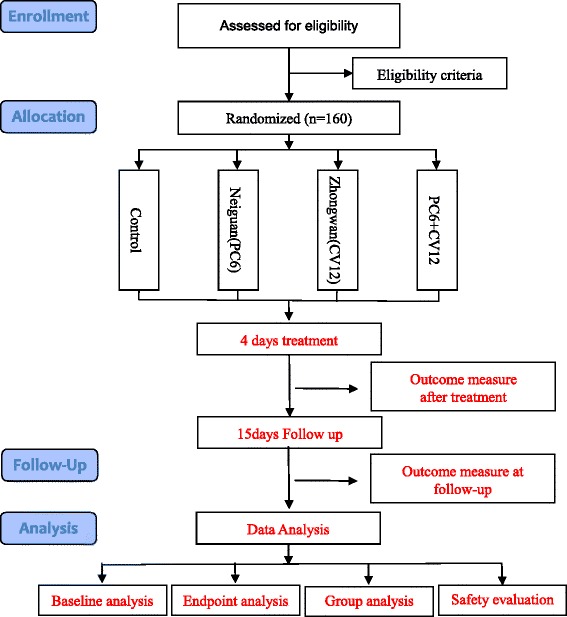
Flow diagram and schedule of enrollment, interventions, and assessments. Assessed for eligibility with eligibility criteria during enrollment, the participants will be randomly divided among four arms: the control group is supplied with a pharmaceutical antiemetic, and the other three arms consist of the antiemetic drug and one type of acupuncture (*Neiguan* (PC6) in one arm or *Zhongwan* (CV12) in another arm), and the antiemetic drug and both types of acupuncture in the remaining arm. The treatment is scheduled to occur within 30 to 60 min before chemotherapy infusion for 4 days. All assessments will be scheduled from the day before chemotherapy to the fifth day of chemotherapy, with follow-up in the next 15 days, followed by data collection and analysis.

All the patients are from the Tianjin Medical University Cancer Institute and Hospital. Our trial started on 14 August 2014, and the first participant was recruited on 18 August 2014. The trial is planned to be completed in one year [Figure 1].

### Interventions

There are four arms in this randomized controlled trial. The control group is supplied with antiemetic pharmaceutical, and the other three arms consist of antiemetic drug and acupuncture.

### Control group

The participants in the control group received standard antiemetic alone. Standard antiemetic for all groups is based on *American Society of Clinical Oncology Clinical Practice Guideline* [7]. The 5-hydroxytryptamine-3 (5-HT3) antagonist (Ramosetron, Tropisetron) and dexamethasone are administered before the chemotherapy treatment.

### Experimental group I: single point PC6

Participants in experimental group I will receive electroacupuncture at the *Neiguan* (PC6) point. The location of *Neiguan* (PC6) is on the anterior aspect of the forearm, between the tendons of the palmaris longus and the flexor carpi radialis, 2 B-cun proximal to the palmar wrist crease [8]. After manipulation until a “*de qi*” sensation is achieved, the needle is connected to an electroacupuncture apparatus. The positive pole is linked to the needle, and the reference pole is secured approximately 1 cm proximal to the acupoint with a plaster. The frequency used is 2/10 Hz, and the intensity of stimulation is adjusted according to the patient’s tolerance, with the electric current set at less than 10 mA. The procedure lasts for 30 min. The treatment is scheduled to occur in the 30 to 60 min before chemotherapy infusion for 4 days.

### Experimental group II: single point CV12

The experimental group II will receive electroacupunture at another single point, *Zhongwan* (CV12). The location of *Zhongwan* (CV12) is on the upper abdomen, 4 B-cun superior to the center of the umbilicus, on the anterior median line [8]. After manipulation until a “*de qi*” sensation is achieved, the needle is connected to an electroacupuncture apparatus. The positive pole is linked to the needle, and the reference pole is secured approximately 1 cm proximal to the acupoint with a plaster. The frequency used is 2/10 Hz, and the intensity of stimulation is adjusted according to the patient’s tolerance, with the electric current set at less than 10 mA. The procedure lasts for 30 min. The treatment is scheduled to occur in the 30 to 60 min before chemotherapy infusion for 4 days.

### Experimental group III: matching points PC6 plus CV12

The experimental group III will receive electroacupuncture at both *Neiguan* point (PC6) and *Zhongwan* point (CV12). After manipulation until a “*de qi*” sensation is achieved, the needle is connected to an electroacupuncture apparatus. The positive pole is linked to the needle, and the reference pole is secured approximately one cm in the proximity of the acupoint with a plaster. The frequency used is 2/10 Hz, and the intensity of stimulation is adjusted according to the patient’s tolerance, with the electric current set at less than 10 mA. The procedure lasts for 30 min. The treatment is scheduled to occur in the 30 min to 60 min before chemotherapy infusion for 4 days.

### Outcomes

#### Primary outcome

The primary outcomes are as follows:

1. The frequency of nausea and vomiting
  Total nausea and vomiting episodes per person over the 6-day study period will be recorded. The frequency of nausea and vomiting is the measurement with the most value for evaluation.
2. The grading of nausea and vomiting
  The Common Terminology Criteria for Adverse Events Version 4.0 [9] will be used to grade the nausea and vomiting.
3. Rhodes Index of Nausea, Vomiting and Retching
  The Rhodes Index of Nausea, Vomiting and Retching [10], an eight-item validated scale, will be used to measure the nausea and vomiting experience, incidence and severity.

### Secondary outcome measures

Secondary outcome measures include the following:

1. The grading of constipation and diarrhea
  The Common Terminology Criteria for Adverse Events Version 4.0 [9] will be used to evaluate the grading of constipation and diarrhea.
2. Electrogastrogram
  Electrogastrogram will be monitored to get the information of gastrointestinal motility.
3. The assessment of quality of life
  The Functional Assessment of Cancer Therapy - General (FACT-G) [11] will be applied to assess the quality of life.
4. The assessment of anxiety and depression
  The Hospital Anxiety and Depression Scale [12] is a scale assessing anxiety with seven items and depression with a further seven items.
5. Other adverse effects during the chemotherapy
  Other adverse effects during the chemotherapy, mainly including constipation and diarrhea, will be assessed by The Common Terminology Criteria for Adverse Events Version 4.0 [9].

### Safety

Any adverse reactions of patient will be recorded in the case report form (CRF), including swelling, pain, bruise at the sites of needle insertion, or discomfort, palpitation, dizziness, *etcetera* after acupuncture.

### Participant timeline

The enrollment will be done before the day of chemotherapy on day zero. The electroacupuncture intervention will be given once daily from day one to day four. All assessments will be scheduled from day zero to day five (see Table 1).

**Table 1:** The schedule of enrollment, interventions, and assessments

	**Study Period**							
	**Enrollment**	**Allocation**	**Post-allocation**					**closeout**
Time point	-t1	0	t1	t2	t3	t4	t5	t6
Enrollment:								
Eligibility screen	×							
Informed consent	×							
Allocation		×						
Interventions:								
Control group								
Experimental group I			×	×	×	×		
Experimental group II			×	×	×	×		
Experimental group III			×	×	×	×		
Assessments:								
baseline variables	×	×						
The frequency of nausea and vomiting		×	×	×	×	×	×	×
The grading of nausea and vomiting		×	×	×	×	×	×	×
Rhodes Index of nausea, vomiting and retching		×	×	×	×	×	×	×
the grading of constipation and diarrhea		×	×	×	×	×		×
electrogastrogram		×					×	×
the assessment of quality of life		×					×	×
the assessment of anxiety and depression		×					×	×
other adverse effect during the chemotherapy							×	×
the adverse effects of acupuncture			×	×	×	×		

t1, the day before the chemotherapy; t1-t5, the first day to the fifth day of chemotherapy; t6, the 21st day of chemotherapy.

### Sample size

Sample size calculation has been based on the result of a trial by *Shen J* [6] and the recommendation of acupuncture specialists in China. The mean value of emesis number in the Neiguan group is 12.5, the standard deviation is 1.5, and the mean value of emesis number in Neiguan plus Zhongwan group is 11, the standard deviation is 1.5. To detect a significant difference between any two groups with a power of 90% and type I error of 5%, the calculated number of patients is 132. Considering a 20% drop-out rate, the total sample size needs 160 patients.

### Allocation and sequence generation

The allocation sequence will be generated by center computer. When the participants voluntarily join this study with informed consents, the practitioner will open the webite (http://iwrs.tice.com.cn/) and evaluate the inclusion and exclusion criteria. If they are accorded with the inclusion criteria, the computer will generate a random number and show which group the participant should be in, and the practitioner will assign the participant to that intervention. The evaluator is blind to which intervention the participant accepts.

### Randomization

The computer-generated randomization is performed remotely by the Evidence-Based Medicine Center in Tianjin on the website of http://iwrs.tice.com.cn/.

### Blinding

The evaluator and the statistician are blinded; neither knows which intervention a participant accepts.

### Statistical methods

Categorical data will be analyzed with the McNemar chi-square test. We will use analysis of variance (ANOVA) to analyze continuous data. If the data trends over time and over time by treatment interactions, we will choose a repeated-measures analysis of variance (ANOVA). A *P* value less than 0.05 is regarded as statistically significant.

### Quality control

All researchers will be required to undergo special training, including trial design, patient inclusion and exclusion criteria, and on filling in the *CRF*. The practitioners have all majored in acupuncture, have an acupuncture degree, and are qualified doctors of Tradition Chinese Medicine. All will have been trained in the standard operating procedure of electroacupuncture. The monitors will check all the case report forms. The reasons of drop-outs and withdrawals during the study will be fully recorded.

#### Ethics

Written informed consent will be obtained from each participant. This study is approved by Medical Ethics Committee-Tianjin University of Traditional Chinese Medicine. The approval number is TJUTCM-EC20140004.

## Discussion

Nausea and vomiting is one of the most common side effects of chemotherapy [13]. Clinical evidence has shown that acupoint stimulation has a certain effect on chemotherapy-induced nausea and vomiting. However, with different interventions used, different results are obtained. Electroacupuncture is effective, and acupressure is protective, while transcutaneous electrical stimulation is ineffective [14]. The effects with the same interventions but different acupoints are different. The objective was to compare acupuncture effects of different acupoint combinations; therefore, there are only a blank group; two, single acupoint groups; and one double-acupoint group, without a sham acupuncture group.

With respect to clinical quality control, the manipulators, estimators and statisticians worked independently to reduce the adverse impacts of artificial factors on data. Although it cannot blind patients or researchers, the estimator and statisticians are unclear about sectionalization. All researchers received pre-work training, and various operation management regulations were formulated to guarantee the consistency of the operation process and evaluation process. A third party has been invited to manage the data independently and design a central randomization system to minimize bias and enhance clinical research quality.

## Trial status

The trial is currently recruiting patients.

## Abbreviations

ALT: glutamic-pyruvic transaminase
AST: glutamic oxalacetic transaminase
BUN: blood urea nitrogen
CINV: chemotherapy-induced nausea and vomiting
Cr: Urine creatinine
CRF: Case Report Form
CV12: The acupoint of Zhongwan
FACT-G: The Functional Assessment of Cancer Therapy - General
KPS: The scoring of Karnofsky
NIH: National Institutes of Health
PC6: The acupoint of Neiguan
ST36: The acupoint of Zusanli
TBIL: total bilirubin
TCM: Traditional Chinese Medicine
